# Antimicrobial photodynamic therapy mediated by phenothiazine photosensitizers against *Candida albicans* and *Candida auris*: a systematic review

**DOI:** 10.1007/s10103-026-05001-4

**Published:** 2026-09-05

**Authors:** Giovanna Fontgalland-Ferreira, Sarah Marinho Figueiredo, Karla Nogueira Matos, Vinicius Leão Roncolato, Claudio Teruo Kassa, Maria Vitória Pigozzi-Portugal, Ilka Tiemy Kato, Renato Araujo Prates

**Affiliations:** 1 Biophotonics-medicine Posgradate Program, Universidade Nove de Julho (UNINOVE), São Paulo, Brazil; 2 Center of Engineering, Modeling and Applied Social Sciences, Federal University of ABC (UFABC), São Bernardo, Brazil; 3 Universidade de São Paulo, São Paulo, Brazil

**Keywords:** Antimicrobial photodynamic therapy, Phenothiazines, *Candida albicans*, *Candida auris*

## Abstract

Fungal infections caused by *Candida albicans* and *Candida auris* represent an increasing clinical challenge, particularly due to biofilm formation and rising antifungal resistance. Antimicrobial photodynamic therapy (aPDT) has emerged as a potential alternative strategy, with phenothiazine-based photosensitizers being among the most extensively investigated compounds. This systematic review aimed to evaluate the application of phenothiazine-mediated aPDT in in vitro studies against *C. albicans* and *C. auris*. A comprehensive search was conducted in PubMed, Embase, and Scopus, including studies published within the last 10 years. Forty in vitro studies met the eligibility criteria and were synthesized descriptively due to substantial methodological heterogeneity. Overall, aPDT was associated with reductions in fungal viability, with generally greater effects reported in planktonic models compared with biofilms. Methylene blue was the most frequently investigated photosensitizer, applied across a broad range of concentrations and dosimetric parameters, resulting in variable antifungal responses. Other phenothiazine derivatives, including toluidine blue O, dimethyl methylene blue, new methylene blue, and S137, were also associated with antifungal activity under specific experimental conditions but remain comparatively underexplored. Studies involving *C. auris* were less frequent and suggested lower susceptibility compared with *C. albicans*, particularly in biofilm models. Given the substantial variability in experimental protocols, especially regarding photosensitizer concentration, light parameters, and biofilm maturation, the findings should be interpreted with caution and limit direct comparison across studies. These findings support the antifungal potential of phenothiazine-mediated aPDT while emphasizing the need for methodological standardization and expanded investigation of *C. auris.*

**Supplementary Information:**

The online version contains supplementary material available at https://doi.org/10.1007/s10103-026-05001-4.

## Introduction

Fungal infections represent a growing clinical challenge, particularly in hospital settings, due to increasing resistance to conventional antifungal agents and the therapeutic limitations associated with their prolonged use [1, 2]. In this context, alternative and adjunctive therapeutic strategies have been explored to overcome microbial resistance mechanisms and expand infection control options, especially for opportunistic fungal pathogens [3–5].

Species of the genus *Candida* are among the leading etiological agents of opportunistic fungal infections. *Candida albicans* remains the most prevalent and widely disseminated species, affecting both mucocutaneous and systemic sites, particularly in immunocompromised and hospitalized patients [1, 6]. Its clinical relevance is largely attributed to its remarkable adaptability, including morphological transitions and biofilm formation, which contribute to infection persistence and reduced susceptibility to antifungal therapy [3, 4]. In contrast, *Candida auris* is an emerging pathogen associated with hospital outbreaks, high mortality rates, and multidrug resistance [1, 7]. Its persistence on hospital surfaces and diagnostic challenges facilitate nosocomial transmission, amplifying its clinical impact despite lower global prevalence [3, 7].

Although pharmacological treatment has been the cornerstone of infection management since the discovery of penicillin [8], the single-target mechanisms of many antimicrobial agents favor the development of resistance over time [9]. Biofilm formation further exacerbates this challenge. Biofilms consist of structured microbial communities embedded in a polymeric matrix that enhances tolerance to antimicrobial agents by limiting drug penetration, supporting persister cell formation, and increasing resistance to high drug concentrations [3, 8, 10, 11]. Moreover, fungal infections are often more difficult to manage than bacterial infections due to the limited number of available antifungal classes and their associated adverse effects [6]. In addition, alternative strategies based on the development of novel antimicrobial compounds targeting key metabolic pathways have also been explored, demonstrating activity against a broad range of microorganisms, including *Candida species* [12, 13].

In parallel with light-based antimicrobial therapies, the development of novel antimicrobial compounds targeting essential metabolic pathways has also been explored as a strategy to overcome antimicrobial resistance. Some of these compounds have demonstrated promising in vitro activity against *Candida spp*.; however, their mechanisms of action differ fundamentally from those of antimicrobial photodynamic therapy, which relies on the light-induced generation of reactive oxygen species. Therefore, these therapeutic strategies should be regarded as complementary rather than competing approaches for the management of fungal infections [12]. Considering these limitations, antimicrobial photodynamic therapy (aPDT) has emerged as a promising alternative with a multi-target mode of action [3, 14]. aPDT involves the administration of a photosensitizer (PS) followed by irradiation with light at an appropriate wavelength, leading to excitation of the PS to a long-lived triplet state [5]. The excited PS interacts with surrounding molecules through type I (electron transfer and free radical formation) and/or type II (energy transfer to molecular oxygen) pathways, resulting in the generation of reactive oxygen species (ROS) and singlet oxygen [2, 5]. These reactive species induce oxidative damage to essential cellular components, including lipids, proteins, and nucleic acids, ultimately leading to microbial inactivation or cell death [5, 10].

The efficacy of aPDT depends on the interaction between the PS and microbial cells, as proximity to cellular targets is essential for effective oxidative damage [10, 15]. Consequently, various photosensitizers have been developed to optimize light absorption properties, singlet oxygen production, and physicochemical characteristics that enhance microbial interaction [15]. Among them, phenothiazine derivatives are particularly relevant [3].

Phenothiazine-based photosensitizers were among the first approved for clinical use and are characterized by their cationic, planar tricyclic structure, which enhances microbial affinity and antimicrobial activity [3, 6]. Methylene blue (MB) and toluidine blue O (TBO), with peak absorption in the red spectrum (approximately 656 nm and 625 nm, respectively), are the most widely investigated compounds in this class [3, 6]. Their absorption at longer wavelengths also favors deeper light penetration into tissues. Despite the expanding body of literature on phenothiazine-mediated aPDT, substantial methodological heterogeneity persists across in vitro studies, particularly regarding photosensitizer concentration, light parameters, pre-irradiation time, and microbial models, limiting reproducibility and direct comparison of findings [3, 5].

Furthermore, most investigations focus on *Candida albicans*, whereas studies involving *Candida auris* remain limited despite its recognized clinical importance and antifungal resistance profile [1, 7]. This imbalance highlights the need for systematic analyses that critically evaluate experimental protocols and reported outcomes.

Therefore, this review aims to systematically evaluate the application of phenothiazine-mediated aPDT in in vitro studies against *Candida albicans* and *Candida auris*, identifying efficacy patterns, frequently employed experimental parameters, and methodological gaps to support future research development.

## Methods

### Study design and reporting guidelines

This study is a systematic review of in vitro studies aimed at evaluating the effects of aPDT against *Candida albicans* and *Candida auris*, with a specific focus on phenothiazine-based photosensitizers.

The review was conducted in accordance with the Preferred Reporting Items for Systematic Reviews and Meta-Analyses (PRISMA) guidelines. A methodological protocol was defined a priori, including eligibility criteria, search strategy, study selection, data extraction, and synthesis procedures, to ensure transparency, reproducibility, and methodological rigor.

### Research question

The research question was structured according to the PICO strategy, adapted for in vitro studies, to guide the search strategy, eligibility criteria, and data extraction.

- **Population (P)**: planktonic cultures or biofilms of *Candida albicans* or *Candida auris*.
- **Intervention (I)**: aPDT using phenothiazine-based photosensitizers.
- **Comparison (C)**: control groups (photosensitizer without light, light without photosensitizer, or negative control).
- **Outcome (O)**: reduction in microbial viability, expressed as log reduction, percentage inhibition, or equivalent quantitative methods.

### Eligibility criteria

To ensure methodological quality and reproducibility of in vitro aPDT studies, essential experimental parameters were defined a priori. Based on these parameters, the following inclusion and exclusion criteria were established.

**Inclusion criteria**:

- In vitro studies involving *Candida albicans* or *Candida auris*.
- Use of aPDT with phenothiazine-based photosensitizers.
- Clear description of essential aPDT parameters.
- Quantitative microbial viability data allowing calculation of log reduction or percentage inhibition.
- Original articles published in peer-reviewed journals within the last 10 years, in English.

**Exclusion criteria**:

Studies were excluded if they failed to clearly and completely report the minimum information required to ensure experimental reproducibility, including:

- In vivo, clinical, or hybrid studies in which aPDT was applied in vivo.
- Non-original articles, including reviews, editorials, protocols, and incomplete reports.
- Studies in which aPDT was not the primary focus but rather a secondary tool for evaluating materials, biomaterials, surfaces, vehicles, devices, or substrates (e.g., resins, polymers, prostheses, or dentures).
- Lack of adequate description of the photosensitizer, particularly the final concentration used.
- Absence of essential light source parameters, including wavelength (nm), preventing protocol reproducibility.
- Inadequate identification of the microbial sample, including species, strain, experimental model (planktonic or biofilm), or culture conditions.
- Absence of appropriate control groups or insufficient data for result comparison.
- Lack of clear description of microbial viability assessment methods, including outcome reporting, number of replicates, or statistical analysis.

### Information sources and search strategy

A comprehensive literature search was conducted in PubMed, Embase, and Scopus to identify in vitro studies evaluating phenothiazine-mediated aPDT against *Candida albicans* and *Candida auris*. The search strategy combined controlled vocabulary and free-text terms related to Candida species, antimicrobial photodynamic therapy, and phenothiazine-based photosensitizers. The final search was conducted in December 2025. Detailed electronic search strategies for each database are provided in Supplementary Table S1.

### Study selection

Study selection was independently performed by three reviewers according to the predefined eligibility criteria. Duplicate records were initially identified using the automatic detection tool in Rayyan and subsequently screened and removed manually by the reviewers to ensure accurate deduplication.

Title and abstract screening was conducted using the Rayyan platform, allowing independent assessment of records. Disagreements between reviewers were resolved by consensus. Articles deemed potentially eligible were subsequently assessed through full-text reading to confirm inclusion.

### Methodological quality assessment

Methodological quality of the included studies was assessed descriptively, considering completeness of reporting of key aPDT experimental parameters, including photosensitizer characterization, light source parameters, microbial model, presence of control groups, and microbial viability assessment methods. This evaluation supported the critical interpretation of results and discussion of methodological heterogeneity among studies, without excluding articles based on quality scoring.

No formal assessment of publication bias or missing outcome bias was performed, as this review was exclusively qualitative and descriptive, with no meta-analysis or quantitative synthesis.

### Data extraction and synthesis

Eligible studies were included in a single qualitative descriptive synthesis. Individual study results were organized into summary tables containing key methodological characteristics and microbiological outcomes to facilitate descriptive comparison across different aPDT protocols.

When necessary, data were standardized to enable descriptive comparison. Photosensitizer concentrations originally reported in mg/L were converted to µM based on the corresponding molar mass when available. Microbiological outcomes were presented as reported in the original studies, prioritizing the primary quantitative outcome (e.g., CFU/mL log reduction or percentage inhibition). Missing data were recorded as “not reported” (NR), without data imputation or additional estimations.

Due to substantial heterogeneity among included studies particularly regarding photosensitizers, concentrations, light parameters, experimental models, and outcome assessment methods no quantitative meta-analysis was performed. Therefore, results were synthesized exclusively through qualitative descriptive analysis, considered the most appropriate approach for integrating the available evidence. Heterogeneity was explored descriptively based on photosensitizer type, microbial model (planktonic cultures or biofilms), and *Candida* species evaluated. No subgroup analyses, meta-regressions, or sensitivity analyses were conducted. Due to variability in outcome measures, results were interpreted descriptively and no direct quantitative comparison across studies was attempted.

## Results

### Study selection

The literature search identified a total of 486 records, including 139 from PubMed, 156 from Embase, and 191 from Scopus. After removal of 274 duplicate records, 212 articles remained for title and abstract screening. At this stage, 155 studies were excluded for not meeting the eligibility criteria.

The remaining 57 articles were assessed through full-text evaluation. During this phase, 17 studies were excluded due to in vivo or animal study design, use of mixed-species biofilms, application of photosensitizers not belonging to the phenothiazine class, or insufficient reporting of essential aPDT parameters, which precluded protocol reproducibility. At the end of the selection process, 40 in vitro studies met the eligibility criteria and were included in the qualitative synthesis.

A detailed flow diagram of study identification, screening, eligibility, and inclusion is presented in Fig. S1.

### General characteristics of included studies

The 40 included studies investigated aPDT mediated by phenothiazine-based photosensitizers against predominantly *Candida albicans*, with a smaller number of studies involving *Candida auris*. Experiments were conducted using both planktonic cultures and biofilm models.

The main methodological characteristics and microbiological outcomes reported in each study are summarized in Table 1.

**Table 1:** Characteristics of the 40 included in vitro studies evaluating phenothiazine-mediated antimicrobial photodynamic therapy (aPDT) against Candida albicans and Candida auris

Study (Author, Year)	Microorganism	Experimental model	Photosensitizer	Light source (nm)	Main outcome
Adnan, 2023 [16]	*C. albicans* (clinical oral isolate)	Suspension	MB	LASER	CFU/mL: 22.6% reduction in the LASER + antifungal group and 45% reduction in the aPDT group.
Bapat, 2021 [17]	*C. auris* (AR0383, AR0389, AR0390)	Biofilm	NMB/TBO	LED	CFU/mL: blue light without FS (inhibition 77%) > red light + FS (≤ 58%) > blue light + FS (≤ 8%).
Brancini, 2016 [18]	*C. albicans* (ATCC 64548)	Suspension	NMB	LED	CFU/mL: reduced survival by 1.6 log₁₀ (3 J/cm²) and 4.6 log₁₀ (10 J/cm²).
Černáková, 2017 [19]	*C. albicans* (SC5314, CY 1123)	Biofilm	MB	LED	CFU/mL: in the presence of fluconazole, 5.89% survived vs. 14.37% without fluconazole; higher aPDT (*P* < 0.01).
Collina, 2022 [11]	*C. albicans* (ATCC 10231)	Biofilm	MB	LED	CFU/mL: reduction of approximately 1.5 log₁₀ after aPDT.
Collina, 2020 [20]	*C. albicans* (ATCC 10231)	Suspension	AzuriA/AzuriB/DMMB	LED	CFU/mL: DMMB was the most effective (1 log₁₀ reduction); aPDT was most effective when FS was delivered in 0.25% SDS.
Collina, 2018 [21]	*C. albicans* (ATCC 10231)	Suspension Biofilm	MB	LED	CFU/mL: light fractionation (10 × 30 s, 2 min in the dark) promoted a reduction of 3.7 log₁₀, representing an improvement of 1.7 log₁₀ compared to continuous irradiation.
Daliri, 2019 [22]	*C. albicans* (ATCC 10231)	Suspension	MB	LASER	CFU/mL: positive control = 201,500 ± 42,093; aPDT showed the lowest number of colonies (log CFU = 3.23 ± 0.37).
Du, 2023 [23]	*C. albicans* (ATCC 90028)	Suspension	MB	LED	CFU/mL: MB + KI-aPDT promoted eradication (~ 7 log₁₀) of *Candida* cells after a single session.
Freire, 2018 [24]	*C. albicans* (ATCC 18804)	Biofilm	MB	LASER	Gene expression: aPDT reduced virulence gene expression (fold-decrease 0.39–0.77 with LASER and 0.43–0.66 with LED), with no difference between the protocols.
Fumes, 2018 [25]	*C. albicans* (ATCC 10231)	Biofilm	MB	LASER	CFU/mL: there was no statistically significant difference between the groups (*p* > 0.05).
Garcia, 2021 [10]	*C. albicans* (SN 425, CNJ 2302, CJN 2330)	Biofilm	TBO	LED	CFU/mL: aPDT applied twice daily reduced biofilm viability by 0.5 log₁₀.
Gonçalves, 2023 [26]	*C. albicans* (ATCC 10231)	Suspension	MB	LED	CFU/mL: in the MB/SDS group, at 26.7 J/cm², reductions ranged from 1–1.8 log, while at 44 J/cm², reductions of 1.7–6.7 log
Hasenleitner, 2019 [27]	*C. albicans* (Mya 273)	Suspension Biofilm	MB	LED	CFU/mL: MB-aPDT photoactivated at 635 nm promoted 99.99% reduction of *C. albicans* with 50 µM MB.
Huang, 2018 [28]	*C. albicans* (SC5314)	Suspension Biofilm	TBO	LED	CFU/mL: aPDT resulted in reductions of 2 log and 1 log in both strains; after aPDT, incubation with fluconazole and posaconazole promoted complete cell death.
Khozeimeh, 2023 [29]	*C. albicans* (ATCC 10231)	Suspension	NMB	LED	CFU/mL: groups nystatin and aPDT + NMB showed the greatest growth inhibitory effects (*P* < 0.001) compared to the control (*P* < 0.05).
Lapena, 2022 [30]	*C. albicans* (ATCC 18804)	Suspension Biofilm	MB	LED	CFU/mL: aPDT caused a reduction of 1.6 log₁₀ in the reference strain and 2.0 log₁₀ in the clinical strain.
Leonel, 2019 [31]	*C. albicans* (ATCC #1023)	Suspension Biofilm	MB	LASER	Viability: in the presence of MB 62.6 µM, biofilm inhibition was 58%, 70%, and 74% with 10, 20, and 30 J/cm², respectively; with MB 15.6–62.6 µM, inhibition ranged from 54–66–55%.
Lin, 2018 [32]	*C. albicans* (SC5314)	Suspension	TBO	LED	CFU/mL: aPDT with TBO promoted a 2–3 log reduction in the viability of *C. albicans*; complete death was observed with 0.25% chitosan.
Loaiza-Toscuento, 2024 [33]	*C. albicans* (CAF2)	Biofilm	MB	LED	Viability: aPDT promoted inhibition and reduction ≥ 98.5% in all strains tested.
Machado, 2023 [34]	*C. albicans* (ATCC 10231)	Biofilm	MB/Azure A/Azure B/DMMB	LED	CFU/mL: aPDT with AA, MB, and DMMB delivered in SDS promoted total inactivation, while AB resulted in a 1.2 log reduction.
Melendez-Celis, 2021 [35]	*C. albicans* (clinical oral isolate)	Biofilm	MB	LED	CFU/mL: in the aPDT group, inhibition of *C. albicans* was > 90% at all doses of fluconazole.
Merigo, 2019 [36]	*C. albicans* (SC5314)	Biofilm	TBO	LASER	Viability: Isolated TBO and aPDT did not show a significant effect, with inhibition of 10–25%.
Nunes, 2024 [37]	*C. albicans* (ATCC 90028)	Suspension Biofilm	DMMB	LED	CFU/mL: aPDT combined with nanotechnology (RL-NPs/DMMB) promoted 99.9% inhibition.
Nunes, 2023 [38]	*C. albicans* (ATCC 90028)	Suspension	DMMB	LED	CFU/mL: aPDT with double application in a single session was the most effective, achieving a 4 log reduction (99.991% inhibition).
Oliveira-Silva, 2019 [39]	*C. albicans* (ATCC 10231)	Suspension	MB	LED	CFU/mL: after 3 min of irradiation, complete cell inactivation occurred (6 log reduction), regardless of the presence of glucose.
Pantyo, 2025 [40]	*C. albicans* (ATCC 10231)	Biofilm	MB	LED	CFU/mL: aPDT reduced growth by 65.8–92.5%.
Pérez-Laguna, 2021 [41]	*C. albicans* (ATCC 10231)	Suspension	MB	LED	CFU/mL: aPDT with isolated methylene blue resulted in a reduction of 6 log₁₀.
Pinto, 2018 [42]	*C. albicans* (ATCC 10231)	Biofilm	TBO	LED	Viability: aPDT with TBO 327 µM promoted ≈ 50% inhibition in biofilm formation.
Rodrigues, 2024 [43]	*C. albicans* (clinical oral isolate)	Suspension	MB	LED	CFU/mL: aPDT promoted a reduction of ≈ 1 log₁₀ with longer irradiation times and higher concentrations.
Rodrigues, 2020 [44]	*C. albicans* (ATCC 64548)	Suspension	NMBN/S137	LED	Viability: at 3 J/cm², S137 was more effective (99.98%; 3.70 log₁₀) than NMBN (85.2%; 0.83 log₁₀); at 9–14 J/cm², both achieved similar mortality rates.
Sabino, 2019 [45]	*C. albicans* (ATCC 90028)	Biofilm	MB	LED	CFU/mL: high tolerance to MB-aPDT (T ≈ 1), with a reduction of 1 log at 10 J and 3 log at 25 J.
Santiago, 2020 [46]	*C. albicans* (ATCC 10231)	Suspension	MB	LASER	CFU/mL: aPDT showed 100% antifungal efficacy.
Silva, 2023 [47]	*C. auris* (CBS 10913)	Suspension Biofilm	MB/DMMB	LED	Viability: aPDT reduced metabolic activity by 87% and biomass by 65%; Biofilm: reductions of 85% and 91%, respectively.
Štefánek, 2022 [48]	*C. auris* (H261, R, S)	Biofilm	MB	LASER	Viability: aPDT was effective in all strains; 300 s of irradiation promoted > 90% inhibition.
Tan, 2019 [49]	*C. auris* (AR382, AR383, AR385, AR386, AR390)	Suspension Biofilm	MB	LED	CFU/mL: Suspension: 100% inhibition at all concentrations; Biofilm: aPDT reduced 1.5–7.20 log₁₀.
Torres-Hurtado, 2019 [50]	*C. albicans* (clinical oral isolate)	Suspension	MB	LED	CFU/mL: aPDT with 5 µM–20 J or 10 µM–10 J promoted 90% inhibition with 3 light exposures; 10 µM–20 J achieved 95% with 2 exposures.
Tunçcan, 2018 [14]	*C. albicans* (ATCC 90028)	Suspension Biofilm	MB	LED	Biomass: aPDT reduced biofilm formation by 45.4% and promoted reductions > 3 log₁₀ in suspension.
Wiench, 2021 [15]	*C. albicans* (ATCC 90.028, ATCC 10.231)	Suspension	TBO	LED	CFU/mL: aPDT promoted a 76.89% reduction after 10 min of pre-incubation.
Wiench, 2019 [51]	*C. albicans* (ATCC 10231)	Biofilm	TBO	LASER	CFU/mL: aPDT reduced from 24 → 0.5, 40.33 → 3.0, and 10.5 → 6.67, depending on time, intensity, and exposure.

MB: methylene blue; TBO: toluidine blue O; NMB: new methylene blue; LED

### Experimental parameters of phenothiazine-mediated aPDT

The included studies applied phenothiazine-based photosensitizers under markedly heterogeneous experimental conditions (Table 2). Despite chemical similarity among compounds, substantial variability was identified in photosensitizer concentration, pre-irradiation time, wavelength, irradiance, and radiant exposure, limiting cross-study comparability. Methylene blue (MB) exhibited the widest concentration range (5–3130 µM), followed by new methylene blue (2.5–3126 µM) and toluidine blue O (10–400 µM), whereas DMMB (1.56–313 µM), Azure A/B (65.4–171 µM), and S137 (2.5 µM) were evaluated within narrower intervals. Pre-irradiation times also varied considerably, particularly for MB (1–60 min) and TBO (1–30 min).

Light delivery was predominantly LED-based, with wavelengths largely confined to the red spectrum (630–660 nm), consistent with phenothiazine absorption profiles. However, a broader wavelength range was reported across studies (450–880 nm), reflecting the emission characteristics of different light sources rather than the effective activation range of methylene blue, which is centered around 660 nm. Some studies also employed wavelengths outside this optimal activation range, including infrared spectra or combined emission profiles.

**Table 2:** Ranges of main aPDT parameters by phenothiazine-based photosensitizers

Parameter	MB	TBO	NMB	Azure A	Azure B	DMMB	S137
Final PS concentration (µM)	5–3130	10–400	2.5–3126	65.4–171	62.6–171	1.56–313	2.5
Pre-irradiation time (min)	1–60	1–30	30	5	5	5–10	30
Light source (number of studies)	LED: 19 LASER: 7	LED: 6 LASER: 2	LED: 4 LASER: 0	LED: 2 LASER: 0	LED: 2 LASER: 0	LED: 5 LASER: 0	LED:1 LASER:0
Wavelength λ (nm)	450–880	450–660	631–660	640–660	640–660	630–662	631–651
Irradiance (mW/cm²)	3–1960	44–192	13–100	2.6–37	2.6–37	2.6–50	13
Dose (J/cm²)	0.3–426.66	12–240	3–240	4.7–60.4	4.7–60.4	4.7–60.4	3–14

MB: methylene blue; TBO: toluidine blue O; NMB: new methylene blue; DMMB: Dimethylmethylene blue

Values are presented as minimum–maximum ranges as reported in the included studies

The symbol “~” indicates approximate values estimated from unit conversions (e.g., mg/L to µM) or approximate

Radiant exposure showed pronounced dispersion, especially for MB (0.3–426.66 J/cm²), reflecting inconsistent dosimetric approaches across studies. Irradiance was not uniformly reported and, when absent, was derived from available parameters. However, incomplete methodological detail particularly omission of beam area or spot size restricted calculation accuracy. Therefore, derived irradiance values should be interpreted cautiously.

Collectively, the wide variability in concentration and light dosimetry parameters highlights the absence of standardized reporting, which may hinder reproducibility and translational progression of phenothiazine-mediated aPDT.

### Antifungal effects in planktonic suspension

In planktonic suspension studies (Table 3), CFU/mL reductions ranged from modest decreases to complete inactivation. Reductions below 3 log₁₀ were observed in different protocols involving MB, NMB, and TBO [15, 16, 20, 29, 31, 43, 50], including reductions of approximately 1 log₁₀ [43], 1.6–2.0 log₁₀ [30], and 2–3 log₁₀ [22].

**Table 3:** Antifungal effects of aPDT in planktonic (Candida) suspension models

Study (Author, Year)	Microorganism	Photosensitizer (µM)	Pre-incubation time (min)	Light source (nm)	Intensity (mW/cm^2^)	Energy density (J/cm²)	Main Outcome
Adnan, 2023 [16]	*C. albicans*	MB; 313	5	LASER; 650	500	150	< 3 log microbial reduction.
Brancini, 2016 [18]	*C. albicans*	NMB; 2.5	30	LED; 631	13.89	3; 10	< 3 log reduction (3 J/cm²) and 4.6 log₁₀ (10 J/cm²).
Collina, 2020 [20]	*C. albicans*	Azure A; 65.4; Azure B; 62.6; DMMB; 1.56	5	LED; 640 ± 12	2.6	4.7	< 3 log microbial reduction.
Collina, 2018 [21]	*C. albicans*	MB: 62.6	5	LED; 640 ± 12	2.6	4.68	3.7 log₁₀ reduction (light fractionation; +1.7 log vs. continuous).
Daliri, 2019 [22]	*C. albicans*	MB; 312.6/625.2	NR	LASER; 660	250	1.5; 25	Lowest CFU (log 3.23 ± 0.37).
Du, 2023 [23]	*C. albicans*	MB; 100–1000	30	LED; 660	50	10–70	~ 7 log₁₀ eradication with aPDT.
Gonçalves, 2023 [26]	*C. albicans*	MB; 62.6	5	LED; 660 ± 10	3.7; 11.2; 18.6; 26.1	4.4; 17.8;	1–6.7 log reduction (dose-dependent).
						26.7; 44	
Hasenleitner, 2019 [27]	*C. albicans*	MB: 10, 20, 50	15	LED; 635	27.6. 6.4 ± 0.5	25.6; 6.6	99.99% inactivation.
Huang, 2018 [28]	*C. albicans*	TBO; 100–400	30	LED; 630	75	50	1–2 log reduction; complete death with antifungal.
Khozeimeh, 2023 [29]	*C. albicans*	NMB; 3126	NR	LED; 660	100	10	Greatest inhibition with nystatin and aPDT.
Lapena, 2022 [30]	*C. albicans*	MB; 300	15	LED: 660	42	30	1.6–2.0 log₁₀ reduction.
Leonel, 2019 [31]	*C. albicans*	MB; 15.6, 31.3, 62.6	10	LASER; 660	263	10; 20; 30	58–74% biofilm inhibition.
Lin, 2018 [32]	*C. albicans*	TBO; 100, 150, 400	30	LED; 630 ± 5	30.1	50	2–3 log reduction with TBO.
Nunes, 2024 [37]	*C. albicans*	DMMB; 313	5	LED; 630 ± 5	41.2	20	99.9% inhibition with RL-NPs/DMMB-aPDT..
Nunes, 2023 [38]	*C. albicans*	DMMB; 2.35	5	LED; 630	41.2	20	4 log₁₀ reduction (99.991%) with double application.
Oliveira-Silva, 2019 [39]	*C. albicans*	MB; 100	10	LED; 660	165	10; 30; 60	after 3 min (6 log₁₀ reduction).
Pérez-Laguna, 2021 [41]	*C. albicans* *C. albicans*	MB; 2000 − 0.094	43, 3.25	LED; 625 ± 10	7; 90	18	6 log₁₀ reduction.
Rodrigues, 2024 [43]		MB; 25, 50, 100	30	LED; 660	45.9	2; 5; 8; 11	~ 1 log₁₀ reduction (dose/time-dependent).
Rodrigues, 2020 [44]	*C. albicans*	NMBN; 2.5, S137; 2.5	30	LED; 631 ± 20	13.89	3; 9; 14	S137 > NMBN at 3 J/cm.
Santiago, 2020 [46]	*C. albicans*	MB; 937	5	LASER; 660	333	426.66	100% antifungal efficacy.
Silva, 2023 [47]	*C. auris*	MB; 12.5, 25, 50, 100 DMMB; 0.375, 0.75, 1.5, 3	10	LED; 662	50	10; 30	87% metabolic and 65% biomass reduction; biofilm 85% and 91%, respectively.
Tan, 2019 [49]	*C. auris*	MB; 25, 50, 100	60	LED; 635	80	12	100% inhibition 1.5–7.2 log₁₀.
Torres-Hurtado, 2019 [50]	*C. albicans*	MB; 5–60	30	LED; 600–650	85.7	10; 60	90–95% inhibition depending on concentration and exposure.
Tunccan, 2018 [14]	*C. albicans*	MB; 78	15	LED; 660	0.78	0.233	< 3 log₁₀ microbial reduction.
Wiench, 2021 [15]	*C. albicans*	TBO; 200	1, 3, 5, 7, 10, 15	LED; 635	800	12	76.9% CFU reduction after 10 min pre-incubation.

Reductions ≥ 3 log₁₀ were reported in multiple studies [18, 21, 23, 26, 27, 38, 39, 41, 49], including protocols employing dual application [38], MB combined with potassium iodide [23], association with SDS [26], light fractionation [21], repetitive irradiation [50], and higher MB concentrations [27, 39]. Complete inactivation (≈ 6–7 log₁₀) was observed under specific conditions [23, 39, 41].

Additionally, some studies reported outcomes as percentage inhibition, demonstrating 90–100% inhibition under certain combinations of photosensitizer concentration and light parameters [46, 50], while others described 100% antifungal efficacy [46, 49]. Overall, greater reductions in suspension were generally associated with higher fluence, increased photosensitizer concentration, and optimized or repeated application protocols.

### Antifungal effects in biofilm model

In biofilm models (Table 4**)**, reductions were generally more moderate compared with planktonic suspension. Reductions below 3 log₁₀ were frequently reported [10, 14, 31, 45, 51], including approximately 0.5 log₁₀ [10], 1–2 log₁₀ [28], and 1–3 log₁₀ depending on the applied fluence [45].

**Table 4:** Antifungal effects of aPDT in. biofilm models

Study (Author, Year)	Microorganism	Photosensitizer (µM)	Pre-incubation time (min)			Light source (nm)	Intensity (mW/cm^2^)	Energy density (J/cm²)	Main outcome
Bapat, 2021 [17]	*C. auris*	NMB; 400; TBO; 400		NR	LED; 620–630, 520–530, 450		32.593; 44.444	176; 240	Red + MB 58–71%, Green + MB 62–76%, Blue + MB 79–84%
Černáková, 2017 [19]	*C. albicans*	MB; 1000		60	LED; 660		190	15; 23; 57	aPDT with fluconazole (5.9% vs. 14.4% survival; *p* < 0.01).
Collina, 2022 [11]	*C. albicans*	MB; 50		5	LED; 640		2.6	4.7	~ 1.5 log₁₀ reduction.
Collina, 2018 [21]	*C. albicans*	MB; 156.3		5	LED; 640 ± 12		2.6	4.68	3.7 log₁₀ reduction with light fractionation.
Freire, 2018 [24]	*C. albicans*	MB; 300		5	LASER; 660		2.6	21.42	Virulence genes downregulated (fold 0.39–0.77).
Fumes, 2018 [25]	*C. albicans*	MB; 312.6		1, 2, 5	LASER; 660		NR	640	No statistically significant difference.
Garcia, 2021 [10]	*C. albicans*	TBO; 44		5	LED; 635		NR	87.6; 175.2	0.5 log₁₀ biofilm reduction (twice-daily aPDT).
Hasenleitner, 2019 [27]	*C. albicans*	MB; 10, 20, 50		15	LED; 635		27.6 ± 2.4; 6.4 ± 0.5	25.6; 6.6	99.99% inactivation (50 µM MB, 635 nm).
Huang, 2018 [28]	*C. albicans*	TBO; 2500		30	LED; 630		75	50	2 log and 1 log reductions; complete killing with azoles.
Lapena, 2022 [30]	*C. albicans*	MB; 600		15	LED: 660		42	30	1.6 log₁₀ (reference) and 2.0 log₁₀ (clinical strain) reduction.
Leonel, 2019 [31]	*C. albicans*	MB; 31.3, 62.6, 156.3		10	LASER; 660		263	10; 20; 30	58–74% inhibition (62.6 µM MB); 54–66% at 156.3)
Loaiza-Toscuento, 2023 [33]	*C. albicans*	MB; 200		30	LED; 630		60	55; 30; 50	≥ 98.5% inhibition in all strains.
Machado, 2023 [34]	*C. albicans*	MB; 156; Azure A; 171; Azure B; 163;DMMB;156		5	LED; 660		37.3	60.4	Total inactivation; 1.2 log reduction with AB.
Melendez-Celis, 2021 [35]	*C. albicans*	MB; 200		NR	LED; 662		9.44	5.6; 11.3; 17; 34	90% inhibition at all fluconazole doses.
Merigo, 2019 [36]	*C. albicans*	TBO; 10		5	LASER; 650		153	10	10–25% inhibition (isolated TBO or aPDT).
Nunes, 2024 [37]	*C. albicans*	DMMB; 313		5	LED; 630 ± 5		41.2	20	99.9% inhibition (RL-NPs/DMMB).
Pantyo, 2025 [40]	*C. albicans*	MB; 3.130		20	LED; 640; 880		5.35	6.42	65.8–92.5% growth reduction.
Pinto, 2018 [42]	*C. albicans*	TBO; 32.7, 65.4, 163.5, 327		5	LED; 630		192.1	21.7	≈ 50% biofilm inhibition (327 µM TBO).
Sabino, 2019 [45]	*C. albicans*	MB; 100		10	LED; 660		100; 38.2	5; 10; 20; 25	1 log (10 J) and 3 log (25 J) reduction; tolerance (T ≈ 1).
Silva, 2023 [47]	*C. auris*	MB; 12.5, 25, 50, 100 DMMB;0.375, 0.75, 1.5,3		10	LED; 662		50	10; 30	87% metabolic and 65% biomass reduction; biofilm 85–91%.
Štefánek, 2022 [48]	*C. auris*	MB; 250, 500, 1000, 2000		15	LASER; 660		190	15; 23; 58	90% inhibition with 300 s irradiation (all strains).
Tan, 2019 [49]	*C. auris*	MB; 25, 50, 100		60	LED; 635		80	12	1.5–7.2 log₁₀ reduction.
Tunçcan, 2018 [14]	*C. albicans*	MB; 78		NR	LED; 660		NR	0. 233	45.4% biofilm reduction.
Wiench, 2019 [51]	*C. albicans*	TBO; 10		1, 3, 5, 7, 10, 15	LASER; 635		NR	12	Time/intensity-dependent reduction (e.g., 24→0.5; 40.3→3.0; 10.5→6.7).

Reductions ≥ 3 log₁₀ were observed under specific conditions involving higher fluence, association with SDS, dual application, or optimized formulations [34, 37, 38, 49, 50]. Tan et al. [49] reported reductions ranging from 1.5 to 7.2 log₁₀ depending on the experimental condition, while Sabino et al. [45] observed a 3 log₁₀ reduction at 25 J. Several studies expressed outcomes as percentage reductions in viability or biomass, frequently ranging from 65 to 90% [40, 47, 48], with some reporting ≥ 98% inhibition [33, 47]. However, these outcomes are not directly comparable to CFU-based log reductions.

Overall, aPDT was associated with antifungal effects in biofilm models, with reductions ≥ 3 log₁₀ occurring less frequently and appearing to depend more on treatment parameters than in planktonic models. Given the heterogeneity in experimental design, light parameters, and outcome assessment methods, these findings should be interpreted descriptively rather than as directly comparable quantitative effects.

## Discussion

The included studies demonstrated substantial methodological heterogeneity in aPDT parameters, experimental models, and outcome assessment, precluding quantitative meta-analysis and supporting a qualitative synthesis. This lack of standardization reflects the absence of standardized experimental frameworks in phenothiazine-mediated aPDT research against *Candida species.* Key parameters, including photosensitizer concentration, pre-irradiation time, wavelength, irradiance, and radiant exposure, varied markedly across studies. For example, methylene blue concentrations ranged from 5 to 3130 µM, while radiant exposure ranged from 0.3 to 426.66 J/cm² substantially limiting direct comparability of outcomes. Therefore, the reported antifungal effects should be interpreted cautiously and within the context of this methodological variability.

Although most protocols employed red wavelengths (630–660 nm), consistent with the absorption peak of phenothiazine-based photosensitizers, minor variations within this range (e.g., 630 nm vs. 640 nm) are likely attributable to device-specific emission characteristics and do not significantly impact photosensitizer activation. However, some studies used alternative spectral ranges that are not optimal for phenothiazine activation. In one study, the light source emitted both red and infrared wavelengths (640 ± 30 nm and 880 ± 30 nm), and since the infrared component lies outside the effective activation range, part of the delivered energy was likely not involved in photodynamic action [40]. Similarly, another study evaluated multiple visible wavelengths (red, green, and blue) in combination with phenothiazines; however, both blue (~ 450 nm) and green (~ 520–530 nm) fall outside the optimal absorption range of these photosensitizers (~ 660 nm), making efficient activation unlikely [17]. Notably, blue light alone demonstrated significant antibiofilm activity in this study, suggesting that its effects may be mediated by endogenous photosensitizers rather than phenothiazine activation. Such deviations from conventional activation parameters may alter photochemical efficiency and complicate the interpretation of antimicrobial outcomes. These findings also highlight the importance of distinguishing the antimicrobial effects produced by endogenous chromophore photoactivation from those mediated by exogenous photosensitizers. Although phenothiazine-based aPDT relies on the absorption of red light, short-wavelength visible light may induce microbial inactivation through the excitation of naturally occurring intracellular chromophores, resulting in reactive oxygen species generation independently of the administered photosensitizer. Consequently, studies employing blue-light irradiation should clearly differentiate these mechanisms by including appropriate control groups and interpreting dosimetric parameters accordingly. Such methodological distinction is essential to improve comparability among studies and to avoid attributing antimicrobial effects exclusively to the photosensitizer when endogenous photoinactivation may also contribute to microbial killing [3, 5, 8, 17].

Additionally, insufficient methodological detail further limited reproducibility, particularly regarding light dosimetry. Irradiance was frequently not explicitly reported and often had to be estimated from available parameters such as output power, irradiation time, and exposure area, however, accurate calculation requires complete information. The absence of these parameters in several studies may introduce uncertainty, and consequently, some estimated values should be interpreted with caution [10, 14, 25, 51]. Similarly, pre-irradiation time was inconsistently reported or not clearly described in multiple studies, making it unclear whether this step was performed [14, 17, 22, 29, 35]. The lack of standardized reporting for these critical variables limits reproducibility and reduces comparability across studies, highlighting the need for more consistent and detailed descriptions of light parameters and experimental conditions in future research.

Several included studies differed in their primary outcomes, reflecting a lack of standardization in the assessment of antifungal activity. While some studies focused on metabolic viability assays [31, 33, 36, 42, 44, 47, 48], others evaluated biofilm biomass [14] or gene expression of virulence factors [24]. These endpoints represent distinct biological processes and are not directly comparable, as metabolic or molecular changes cannot be interpreted as equivalent to fungicidal activity measured by CFU reduction. Therefore, when available, CFU-based data were used for comparison in the present review, as they represent the most standardized and comparable measure of microbial viability. This approach allowed for greater consistency across studies; however, differences in experimental design and outcome measures likely contributed to the heterogeneity observed, and comparisons across studies should be interpreted with caution and considered primarily descriptive rather than strictly quantitative.

Across models, both planktonic suspensions and biofilms demonstrated relevant susceptibility to aPDT, although differences in response were observed depending on experimental conditions. In planktonic models, several studies reported reductions around or exceeding 3 log₁₀ [16, 18, 20, 21], with some cases reaching up to 6 log₁₀ reduction or complete inactivation [38, 41]. Biofilm models also exhibited meaningful antimicrobial effects; however, the magnitude of reduction varied across studies, with some reporting more modest decreases (e.g., 0.5–2 log₁₀ or below 50% inhibition) [10, 30, 36, 42], while others demonstrated more substantial reductions depending on the applied conditions. This variability may be explained by the structural and physiological characteristics of biofilms, including the presence of an extracellular polymeric matrix, which can act as a physical and chemical barrier, limiting photosensitizer penetration and light diffusion. In addition, cells within biofilms often exhibit altered metabolic states and increased tolerance to stress. Furthermore, differences in biofilm time across studies may also have influenced treatment outcomes, as more mature biofilms tend to present greater structural organization and resistance, contributing to variability in the observed responses.

Although most investigations focused on *Candida albicans*, only a limited number of studies evaluated *Candida auris*, including Bapat et al. [17], Silva et al. [47], Štefánek et al. [48], and Tan et al. [49]. Despite demonstrating relevant susceptibility to aPDT under both planktonic and biofilm conditions, particularly in studies reporting high reductions, the small number of available investigations limits robust comparisons between species. Given the multidrug-resistant profile and clinical relevance of *Candida auris*, this represents an important gap in the literature and highlights the need for further targeted studies under standardized conditions.

In addition to the intrinsic resistance of biofilms, variations in experimental parameters appear to play a significant role in treatment outcomes. Greater antimicrobial effects were frequently associated with longer pre-irradiation times, generally exceeding 10 min, suggesting that increased photosensitizer uptake may enhance aPDT efficacy. Similarly, longer irradiation times were associated with improved outcomes in some studies, as observed by Štefánek et al. [48]. In contrast, no consistent relationship was observed between energy density and antimicrobial efficacy, as substantial reductions were achieved even at lower energy doses. Differences in photosensitizer concentration also appeared to influence outcomes, with higher concentrations generally resulting in greater reductions [42].

Overall, these findings indicate that aPDT efficacy is influenced by a combination of biological and experimental factors rather than a single dosimetric parameter. While planktonic models consistently demonstrate high susceptibility, biofilm-associated tolerance remains a major challenge, requiring optimization of treatment parameters. The variability observed across studies underscores the complexity of translating in vitro findings into standardized protocols and highlights the importance of methodological consistency in future research.

## Conclusion

Phenothiazine-mediated antimicrobial photodynamic therapy was associated with in vitro antifungal activity against *Candida albicans* and *Candida auris*, with a tendency toward greater susceptibility in planktonic models compared with biofilms. However, substantial heterogeneity in dosimetric parameters and experimental design limits direct comparison across studies. Standardized protocols and rigorous methodological reporting are essential to support reproducibility and advance the translational development of aPDT as a complementary antifungal strategy.

## Supplementary Information

Below is the link to the electronic supplementary material.

Supplementary Material 1

Supplementary Material 2

## Data Availability

No datasets were generated or analysed during the current study.
