# Supplementary material for: Antimicrobial photodynamic therapy mediated by phenothiazine photosensitizers against Candida albicans and Candida auris: a systematic review
Source: Lasers Med Sci. 2026 Sep 5;41(1):214. doi: 10.1007/s10103-026-05001-4 (PMC13546162; doi:10.1007/s10103-026-05001-4)
Supplement: Supplementary file 1 [file 10103_2026_5001_MOESM1_ESM.docx]

**Supplementary Table S1. Electronic search strategies and number of records retrieved from each database.**

| **Database** | **n** | **Search Strategy** |
| --- | --- | --- |
| Embase | 156 | (('candida albicans'/exp OR 'candida auris'/exp OR 'candida albicans':ti,ab OR 'candida auris':ti,ab) AND ('photochemotherapy'/exp OR 'photodynamic therapy'/exp OR 'photochemotherapy':ti,ab OR 'photodynamic therapy':ti,ab OR 'aPDT':ti,ab OR 'photodynamic inactivation':ti,ab OR 'photoinactivation':ti,ab) AND ('phenothiazine'/exp OR phenothiazine*:ti,ab OR 'methylene blue':ti,ab OR 'toluidine blue':ti,ab OR 'TBO':ti,ab OR 'MB':ti,ab OR 'BuTBO':ti,ab OR 'new methylene blue':ti,ab OR 'azure A':ti,ab OR 'azure B':ti,ab OR 'S137':ti,ab OR 'dimethyl methylene blue':ti,ab) NOT ('clinical trial'/exp NOT 'animal model'/exp)) |
| Pubmed | 139 | (("Candida albicans" OR "Candida auris") AND ("Photochemotherapy" OR "Photodynamic Therapy" OR "aPDT" OR "photodynamic inactivation" OR "photoinactivation") AND ("Phenothiazines" OR phenothiazine* OR "methylene blue" OR "toluidine blue" OR "TBO" OR "MB" OR "BuTBO" OR "new methylene blue" OR "azure A" OR "azure B" OR "S137" OR "dimethyl methylene blue") NOT ("clinical trial" NOT "Animal Model")) |
| Scopus | 191 | TITLE-ABS-KEY("candida albicans" OR "candida auris") AND TITLE-ABS-KEY("photochemotherapy" OR "photodynamic therapy" OR "aPDT" OR "photodynamic inactivation" OR "photoinactivation") AND TITLE-ABS-KEY("phenothiazine" OR "methylene blue" OR "toluidine blue" OR "new methylene blue" OR "S137" OR "azure A" OR "azure B" OR "dimethyl methylene blue" OR "TBO" OR "MB" OR "BuTBO" OR "butyl toluidine blue" OR "butylated toluidine blue") AND NOT (TITLE-ABS-KEY("clinical trial") AND NOT TITLE-ABS-KEY("animal model")) |
