# Supplementary material for: Antimicrobial photodynamic therapy mediated by phenothiazine photosensitizers against Candida albicans and Candida auris: a systematic review
Source: Lasers Med Sci. 2026 Sep 5;41(1):214. doi: 10.1007/s10103-026-05001-4 (PMC13546162; doi:10.1007/s10103-026-05001-4)
Supplement: Supplementary file 2 [file 10103_2026_5001_MOESM2_ESM.docx]

**Fig. S1. PRISMA flow diagram of study identification, screening, eligibility, and inclusion.**

**Identification of studies via databases and registers**

**Identification**

Records identified from*:

Databases (n = 486)

PubMed (n = 139)
Embase (n = 156)
Scopus (n = 191)

Records removed *before screening*:

Duplicate records removed (n = 274) using Rayyan
Records marked as ineligible by automation tools (n = 0)

Records screened

(n = 212)

Records excluded**

(n = 155)

**Screening**

Reports sought for retrieval

(n =57)

Reports not retrieved

(n = 0)

Reports excluded:17

Animal or *in vivo* model (n = 4)

Mixed-species biofilm model

(n = 7)

Not phenothiazine-based photosensitizer (n=2)

Insufficient reporting of photodynamic parameters

(n = 4)

Reports assessed for eligibility

(n =57)

Studies included in review

(n =40)

**Included**
